# Volumetric Evaluation of Alveolar Ridge Preservation Using Dense PTFE (d-PTFE) Membranes Intentionally Exposed to the Oral Cavity After Immediate Implant Placement—A Retrospective Study

**DOI:** 10.3390/dj13020058

**Published:** 2025-01-27

**Authors:** Ulisses R. C. Dayube, Thabet Asbi, Marcio Formiga, Eduardo Groisman, João Paulo Bortoli, Fernando G. Lima, Yaniv Mayer, Doron Haim, Isabelle Meinster, Eran Gabay, Jamil A. Shibli

**Affiliations:** 1 Department of Periodontology, Dental Research Division, Guarulhos University, Guarulhos 07023-070, Brazil; 2 Department of Periodontology and Implant Dentistry, Rambam Health Care Campus, Haifa 3525408, Israel; 3 Maccabi-Dent Research Department, Tel-Aviv 6801298, Israel; 4 Department of Periodontology and Oral Implantology, University of Vale do Itajaí, São José 88302-901, Brazil; 5 Rappaport Faculty of Medicine, Technion institute of technology, Haifa 3200003, Israel

**Keywords:** immediate implants, guided bone regeneration, PTFE membranes, biomaterials

## Abstract

**Background:** Tooth loss causes alveolar bone resorption, which may hinder the ability of implant placement. Socket preservation with immediate implant placement is one of the methods used to reduce bone resorption. In this retrospective study, we evaluated the influence of the use of dense polytetrafluoroethylene (d-PTFE) membranes on alveolar preservation after tooth extraction and with the installation of immediate dental implants. **Methods:** In this retrospective study, one hundred and four patients were divided into two groups: immediate implant and gap filling with heterogenous bone graft (control group, 52 patients) or immediate implant, gap filling with heterogenous bone graft, and covering with a d-PTFE membrane with dimensions of 12 × 24 mm, which was intentionally left exposed to the oral cavity (test group, 52 patients). Tomographic data were obtained before and 12 months after the surgical procedures. **Results:** The membranes exposed in the oral cavity showed no infection. Volumetric analyses revealed a statistically significant difference in alveolar ridge resorption for the control and d-PTFE groups, 16.75% and 4.55%, respectively. **Conclusions:** Intentionally exposed d-PTFE membranes showed minimal complications. Based on the volumetric results, alveolar ridge preservation with d-PTFE membranes was superior to the bone graft alone in immediate implant placement.

## 1. Introduction

Tooth loss not only impacts the function and esthetics of the oral cavity but also triggers significant alterations in the alveolar ridge’s bone structure and surrounding soft tissues [1]. These changes, particularly pronounced in the buccal region, present challenges for prosthetic rehabilitation by compromising both implant placement and esthetic results.

Schropp et al. (2003) demonstrated that these changes can reach up to 30% in the buccolingual direction within three months and up to 50% after the first year [2]. The most significant changes occur within the first two months, with more pronounced resorption in the buccal aspect [2].

Various strategies have been explored to address these challenges, including socket preservation, immediate implant placement, and the use of biomaterials like membranes and bone grafts. Numerous techniques have been proposed to preserve or reduce volumetric changes in the alveolar ridge following tooth extraction. These include minimally invasive extractions, flapless procedures, immediate implant placement, and socket preservation with or without the use of membranes [3,4,5,6,7,8,9]. In 2001, Paolantonio et al. suggested that immediate implant placement would prevent bone remodeling and maintain the original shape of the ridge [10]. However, this was challenged in 2004 by Botticelli et al., who conducted a study on immediate implants and found a 50% reduction in the buccal aspect and 25% in the lingual aspect. This aligns with Araujo et al.’s studies on dogs in 2005 and 2006 [11].

Immediate implant placement following tooth extraction generally yields predictable outcomes, provided there is sufficient residual bone to achieve primary implant stability [10]. This approach offers several advantages, including a reduction in the number of surgical procedures, preservation of the peri-implant soft tissue architecture, and a shortened overall treatment duration [12,13,14,15]. When supplemented with socket grafting, immediate implant placement can better sustain bone architecture, particularly on the buccal wall, critical for optimal pink esthetics [16,17]. The impact of flap elevation has also been explored in the literature. Araujo (2009a), in a study on dogs, reported no statistically significant difference in bone remodeling between groups with or without full-thickness flap elevation [18]. However, Fickl et al. (2008) presented contrasting findings [19]. A similar outcome was observed in a human study conducted by Novaes et al. (2012), which showed that the flap-elevation group experienced double the bone loss compared to the non-flap group [20]. Socket preservation was suggested to prevent bone remodeling. Among the biomaterials used in socket preservation are xenografts, which have been shown to be effective in preserving bone in several studies [8,9]. De Calvelho Formiga et al. (2019) concluded that using xenografts combined with dense polytetrafluoroethylene (d-PTFE) membranes proved superior to using the same d-PTFE membrane and blood clots alone in post-extraction sockets [21].

The first membranes used were made of expanded polytetrafluoroethylene (e-PTFE), with or without titanium support [22]. This material is inert and does not cause tissue reactions when inserted into the human body. Studies demonstrate the effectiveness of using non-resorbable membranes in the regeneration of bone defects in animals and humans [7,22,23,24].

In 2001, Bartee reported that the low porosity of PTFE membranes (<0.2 μm) results in a high surface density, effectively preventing bacterial infiltration [25]. This characteristic allows the membrane to be left exposed in the oral cavity with minimal risk of infection and graft failure. Additionally, the membrane’s ability to remain exposed eliminates the need for extensive flap designs and releasing incisions to achieve primary closure [23,25] (Bartee, 2001; Barber et al., 2007). Consequently, this approach helps preserve the natural architecture of keratinized gingival tissue and the interdental papilla in their original positions [23,25].

Bartee (1998) reported that the optimal timing for membrane removal depends on the defect size and degree of vascularization, ensuring proper bone formation. Removing the membrane between 21 and 28 days permits the blood supply from the upper portion of the defect to adequately support bone development [7].

Numerous clinical cases involving humans have utilized the d-PTFE membrane for ridge preservation following single or multiple tooth extractions, with both delayed and immediate implant placements. All cases yielded satisfactory outcomes. Histological analysis revealed dense connective tissue with a clinical texture akin to osteoid. The implants were fully embedded in the bone tissue [21,23,26].

The high predictability of the d-PTFE membrane in ridge-preservation procedures makes it a reliable option for routine use in post-extraction [27] and post-explantation sites [28]. There is still limited understanding of the effectiveness of certain interventions, especially regarding the intentional exposure of d-PTFE membranes to the oral environment during immediate implant placement. To our knowledge, the impact of d-PTFE membranes on volumetric changes in the alveolar ridge associated with immediate implant placement has not been explored in the existing literature.

The objective of this retrospective study was to tomographically evaluate the volumetric changes in the alveolar ridge after socket preservation in post-extraction sites with immediate implant placement and xenogenous bone grafting, with or without the use of a d-PTFE membrane intentionally exposed to the oral environment.

## 2. Materials and Methods

### 2.1. Study Registration and Design

This retrospective case–control study was conducted at the Postgraduate Implantology Clinic of the University of Guarulhos—SP. The study received approval from the university’s Institutional Ethics Committee (approval number 203/13 November 2017). It was carried out by the ethical principles outlined in the Declaration of Helsinki and the Ethical Guidelines for Research Involving Human Subjects. All participants provided informed consent to use their data in the study. Personal data were securely stored on the clinic’s computer system to protect patient confidentiality, and were accessible only through a confidential access code.

### 2.2. Inclusion Criteria

**Figure 1:**
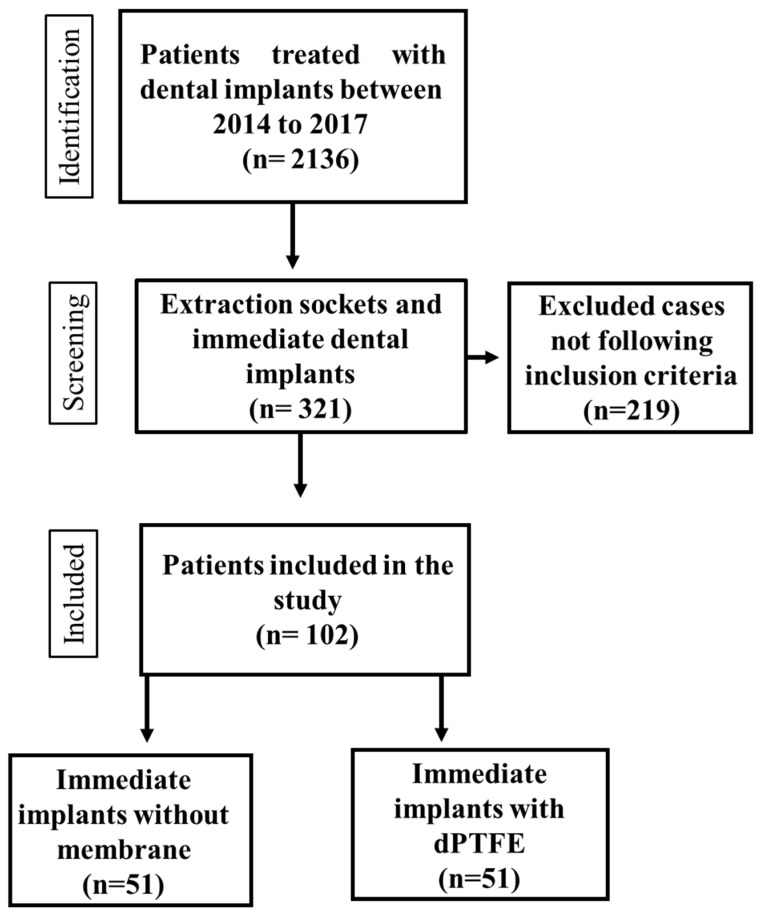
Flowchart of the study design.

Patients over 18 years of age who underwent extraction of a single tooth, intending to replace it with a dental implant, who were non-smokers, systemically healthy, and who signed an informed consent to use their data and attended the 12-month follow-up visit for the final tomographic re-evaluation procedures were included in this study (Figure 1).

### 2.3. Exclusion Criteria

Cases involving lower incisors, reports of allergies to any of the products used in the study, smoking patients, uncontrolled diabetes, hematological disorders such as hemophilia or leukemia, local or systemic infections that could compromise healing, hepatic or renal dysfunction or insufficiency, patients undergoing cancer treatment or having undergone radiotherapy or chemotherapy within the last 18 months, history of oral bisphosphonate use, pregnant women, cases diagnosed with active periodontitis, and patients with missing data in their files were excluded from the study. Patients with reduced periodontium were also excluded.

All patients were required to read, understand, and sign an informed consent form.

### 2.4. Treatment Groups

The selected patients included in this study received one of the two treatment types.

**Figure 2:**
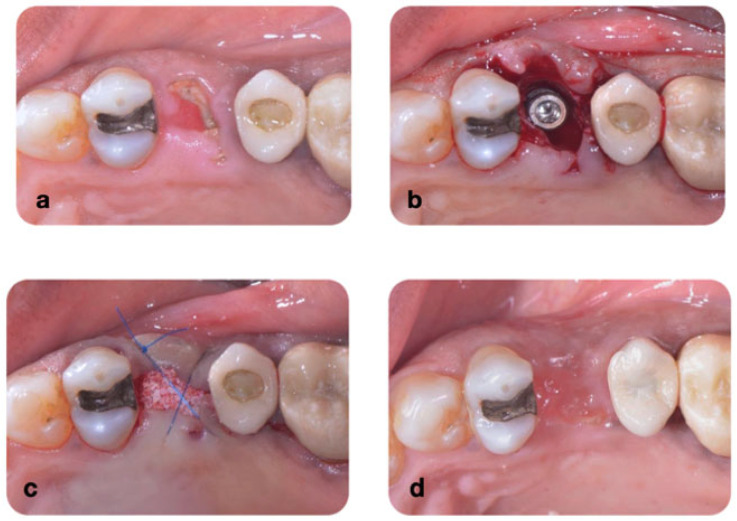
(**a**) Fractured tooth; (**b**) immediate implant placement; (**c**) gaps filled with xenograft material sutured with polypropylene 4.0; (**d**) occlusal view of the area after 12-month healing period. There was buccal loss after the healing period.

Control group (n = 51 subjects): Tooth extraction, immediate implant placement (IMPLACIL DE BORTOLI, São Paulo, Brazil), gap filling with a bovine-derived bone substitute (Lumina Pourus, Critéria, São Paulo, Brazil), and suturing with polypropylene thread (Polypropylene Thread 4-0, 17 mm needle ½—Bioline, Anápolis, GO, Brazil) without covering with any type of membrane (Figure 2).

**Figure 3:**
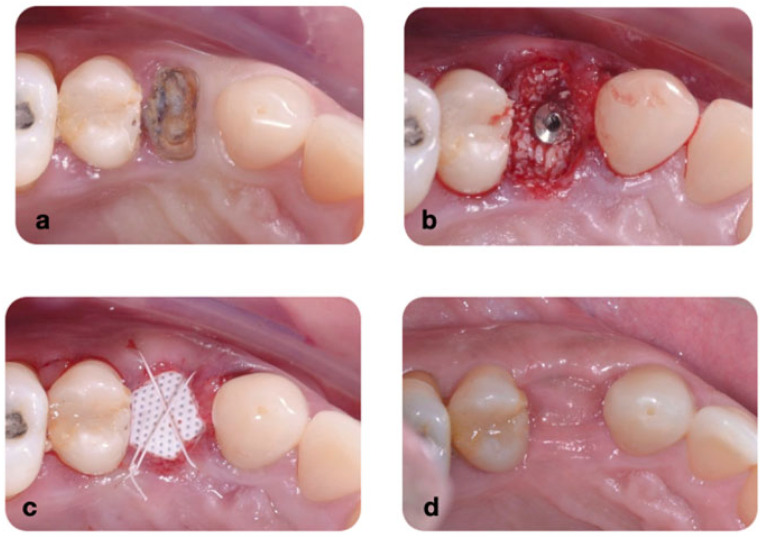
Clinical view of the surgical procedures: (**a**) fractured tooth; (**b**) immediate implant placement with the gaps filled with xenograft material; (**c**) intentionally exposed d-PTFE suture with Teflon 4.0; (**d**) occlusal view of the area after 12-month healing period. Note that there was no buccal loss after the procedure.

Test group (n = 51 subjects): Tooth extraction, immediate implant placement (IMPLACIL DE BORTOLI, São Paulo, Brazil), gap filling with a bovine-derived bone substitute (Lumina Pourus, Critéria, São Paulo, Brazil), installation of d-PTFE membrane (Cytoplast TXT-200 Singles, Osteogenics Biomedical, Lubbock, TX, USA), and d-PTFE suturing (CS051819 d-PTFE Thread 3-0, 19 mm needle 3/8 Cytoplast, Osteogenic Biomedical, Lubbock, TX, USA) (Figure 3).

### 2.5. Surgical Procedures, Postoperative Care, and Follow-Up Appointments

Before each surgical intervention, a cone beam computed tomography (CBCT) scan (i-CAT, Imaging Sciences International, Hatfield, PA, USA) was performed on the region of interest to minimize radiation exposure. The field of view was 6 cm, and the machine settings were fixed at 120 kVp and 18.66 mAs for all scans.

**Figure 4:**
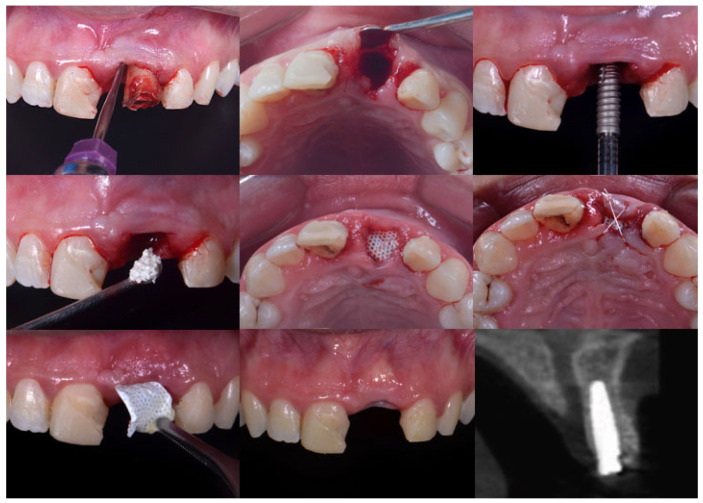
Clinical sequence of the surgical and clinical steps: the tooth extraction; an inspection of the alveoli; immediate implant placement; xenograft material placed on the surgical site; d-PTFE intentionally exposed; suture with Teflon; membrane removal after 21 days; and final clinical and radiographic aspect of the area.

Patients were prescribed 2 g of amoxicillin to be taken 1 h before the surgical procedure and continued with 500 mg 3 times a day for the following 5 days. For patients with an allergy to penicillin, 500 mg of azithromycin was prescribed, with one tablet to be taken daily for 5 days, starting 1 h before the surgical procedure. Initially, patients were instructed to rinse with 0.12% chlorhexidine for 1 min. Extraoral decontamination was performed with 2% chlorhexidine. One experienced surgeon treated all cases. Surgical procedures were performed under local anesthesia with 4% articaine with 1:100,000 epinephrine (DFL, Santa Catarina, Brazil). Tooth extraction was carefully performed using manual microsurgery instruments to minimize damage to the gingiva (Figure 3) and the underlying alveolar bone. After extraction, the sockets were gently curetted. Drilling was performed according to the manufacturer’s guidelines. Then, an implant was placed in the extraction socket. The gap between the implant and the socket walls was filled with xenogeneic bone. Group 1 patients received only polypropylene sutures. In the test group (d-PTFE), patients underwent the same surgical procedure, and then the socket was covered with a d-PTFE membrane, and PTFE sutures were applied (Figure 4).

Anti-inflammatory medication (600 mg of ibuprofen) every 6 h for 5 days and mouthwash (0.12% chlorhexidine) 3 times daily for one week were prescribed. One week postoperatively, sutures were removed, and oral hygiene instructions were reinforced. For the PTFE group patients, a cotton swab soaked in 0.12% chlorhexidine was used to clean the exposed d-PTFE membrane gently. In the test group, d-PTFE membranes were removed after 18 to 22 days with clinical tweezers under topical anesthesia. After 12 months, the sites were examined again, and new CBCT scans were performed.

### 2.6. Volumetric Analyses

**Figure 5:**
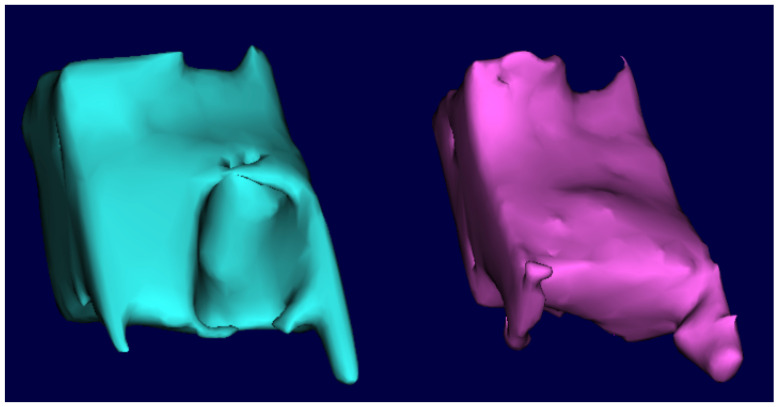
Segmentation of the ROI before and after 12-month healing.

**Figure 6:**
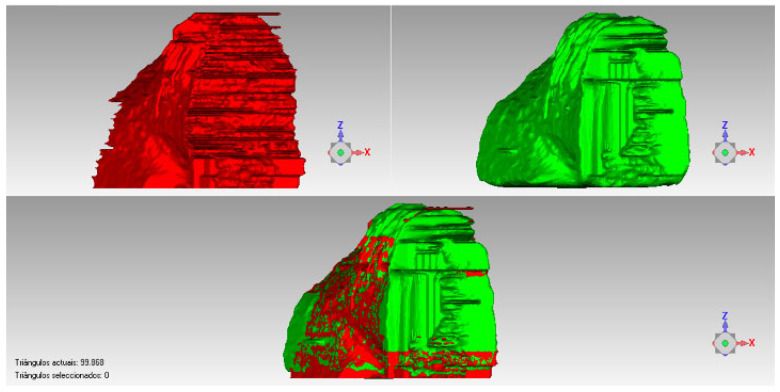
An example of the ROI evaluation; lower image shows the above images overlapping.

The DICOM (Digital Imaging and Communications in Medicine) files were uploaded into the InVesalius software (version 3.1.1, CTI Renato Archer, Campinas, Brazil), which allowed the selection of a region of interest (ROI). The segmentation settings of the selected ROI from the initial scan were applied to the 12-month scan to generate comparable volumes (Figure 5). The ROI included the entire bone area mesiodistally, with its borders extending to the two adjacent teeth. Apicocoronally, the ROI encompassed 4 mm beyond the extraction socket. Therefore, the apical and proximal areas aligned both images to reduce the possible bias during the alignment and measurements. Software (version 2024.1.0, Geomagic Control X, 3D Systems, Rock Hill, SC, USA) was used to quantify the volume of each ROI. An examiner who was blinded to the treatment group (test or control) then calculated the volumetric changes from baseline to 12 months using subtraction analysis, expressed in percentage values (Figure 6).

### 2.7. Statistics

Data analysis in this retrospective study was performed using descriptive statistics for the volumetric results. Power calculation was set considering a 15% decrease in bone loss in the approximal area of the test group compared to the control group; the significance level was set as 0.05 and the power as 80%, resulting in at least 46 subjects being enrolled in each group. A non-parametric Mann–Whitney rank test (GraphPad Prism 10 (version 10.4.0, GraphPad Software, San Diego, CA, USA).) was used to evaluate significant differences between the effects of different treatment types at 12 months. A *p*-value < 0.05 was considered statistically significant.

## 3. Results

### 3.1. Study Population and Sites

One hundred and two individuals (28 men and 74 women) met the inclusion criteria for this retrospective study (n = 51 patients per group). The patients’ ages ranged from 22 to 65 years (average age: 33.5 years). Only one tooth per patient was treated. All the teeth included in this study had a well-preserved periodontium (no interproximal bone loss > 2 mm). The distribution of the teeth was as follows: 5 upper central incisors, 9 upper lateral incisors, 2 upper canines, 32 upper premolars, 17 upper molars, 2 lower lateral incisors, 20 lower premolars, and 15 lower molars. The reasons for extraction included fractures (n = 45), extensive caries (n = 26), endodontic failure (n = 21), and extensive root resorption (n = 10) (Table 1). There were no differences between the groups (*p* > 0.05).

### 3.2. Clinical Outcomes

Healing proceeded without complications in all cases, and all patients remained in the study until the completion of their prosthetic treatments. In the test group, the membranes demonstrated excellent biocompatibility, with no indications of inflammation or infection. d-PTFE membrane removal occurred between 18 and 22 days post-surgery.

### 3.3. Volumetric Results

**Figure 7:**
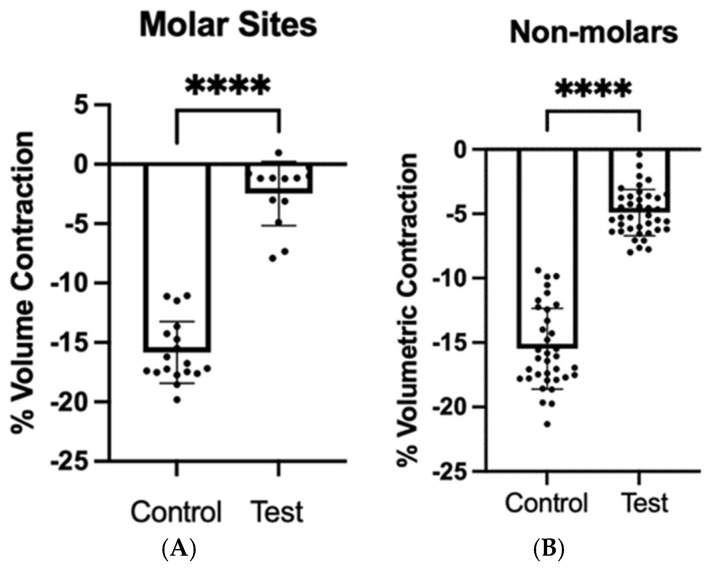
Scatter dot plot with a mean (±SD) of the volume contraction of the alveolar sites for control and test (d-PTFE) groups after 12-month follow-up for (**A**) molar and (**B**) non-molar sites, respectively. Mann–Whitney Test (**** = *p* < 0.0001).

Volumetric analyses revealed significantly different resorption rates of the alveolar ridge between the control and test groups, with resorption values of 16.41% and 4.55%, respectively, as shown in Table 2 (*p* < 0.0001). When the teeth were divided into molars and non-molars (Figure 7), the test group showed lower volumetric contraction when compared with the control group (*p* < 0.0001).

## 4. Discussion

This retrospective study evaluated the effects and performance of d-PTFE membranes when exposed to the oral cavity following tooth extraction and immediate implant placement. Our findings indicate that the use of d-PTFE membranes, even when internationally left exposed to the oral environment, did not lead to any complications or infections, and inflammation was minimal. Notably, the membrane significantly reduced bone resorption, showing a fourfold decrease compared to the use of bone grafts alone.

Several studies have reported comparable outcomes, supporting the efficacy of dense PTFE membranes in similar clinical contexts, whether used alone or in conjunction with other biomaterials [21,23,26]. For instance, de Carvalho Formiga et al. (2019) demonstrated PTFE membranes’ favorable biocompatibility and long-term stability, particularly in preserving alveolar bone height post-extraction [21]. Similarly, Ronda et al. (2014) found that the use of PTFE membranes contributed to superior bone preservation and implant stability compared to other grafting techniques [29]. Barboza et al. (2010) further corroborated these findings, highlighting that d-PTFE membranes effectively minimize soft tissue interference, especially avoiding flaps and incision to provide soft tissue closure by first intention, and promote osseointegration, a crucial factor in the success of immediate implants [30]. Finally, Dayube et al. (2017) reinforced that d-PTFE membranes, whether combined with other biomaterials or as a standalone barrier, can effectively mitigate bone loss, support soft tissue healing, and reduce the risk of postoperative complications [28].

Taken together, these studies and our findings underscore the reliability and effectiveness of d-PTFE membranes in immediate implant placement procedures. Their ability to provide robust bone preservation and minimal complications positions them as a valuable tool in clinical practice for optimizing esthetic and functional outcomes.

d-PTFE membranes have proven to be safe for exposure to the oral environment and provide a high level of predictability in preserving bone volume [31]. In some cases, the removal of these membranes may cause minor bleeding, indicating a biological attachment to the membrane surface [31]. This cellular adhesion is crucial, as it helps create a seal around the edges of the exposed d-PTFE membranes, which is essential for maintaining primary closure, especially in more extensive grafting procedures [2,5,28,30].

This study showed that leaving the membrane exposed to the oral cavity is safe and does not cause any complications. Consequently, large flaps and horizontal releasing incisions are not necessary to perform primary closures of the extraction socket in certain clinical situations, yet they provide coverage of the particulate graft materials, preserving the architecture of the soft tissues and maintaining the width of the keratinized tissue [28,30,32].

Another advantage of d-PTFE membranes is that their high density facilitates simple membrane removal, avoiding second surgery [31,32]. In this study, the membranes were easily removed without damaging the newly formed underlying tissues or causing discomfort to the patient.

According to some authors, while d-PTFE is easy to remove, e-PTFE requires a more complicated reentry surgery, likely due to fibrointegration into the membrane’s porosities [2,29,30].

Most of the studies align with our findings, which showed that exposure of the d-PTFE membrane to the oral environment does not result in contamination of the treated site, thus not necessitating complete soft tissue coverage [24,28,29,33,34].

Bovine-derived xenogenous bone substitutes are already widely known and used for filling gaps between the implant and the remaining bone walls or simply in alveolar bone regeneration [9,13,18,21]. In our study, they were used in both groups, with immediate implant placement, to ensure that only one variable was analyzed.

In this study, the bone changes were minimal for the control and d-PTFE groups (16.75% and 4.55%, respectively). These changes are lower than the results presented by Sanz et al., which showed resorption of 29% of the grafted group; this could be attributed to the fact that in their research, the thickness of the buccal bone was less than 1 mm [35]. However, other authors reported similar results of 16% resorption in the grafted group [36,37].

This retrospective case–control study has several limitations. While only the lower central incisors were excluded due to their small root diameters and corresponding smaller socket sizes, the inclusion of upper molars and lateral incisors—each with significantly different root diameters—may have introduced variability, potentially affecting the outcomes by influencing distinct resorption patterns. Additionally, combining data from both maxillary and mandibular arches, which possess differing anatomical characteristics such as variations in cortical bone thickness and the quantity of medullary bone surrounding the teeth, may have contributed to further discrepancies. Our study could not precisely provide information on the site (approximal or proximal), although some clinical pictures depicted that buccal sites were more prone to present resorption than lingual sites. Finally, regression analysis did not evaluate our data to avoid the influence and bias of some confounding factors.

Another limitation is the absence of a control group utilizing a resorbable membrane, which could have simplified the procedure. However, numerous studies have already examined the use of resorbable membranes [9,38] and the objective of this study was to investigate the potential complications and benefits of d-PTFE membranes when exposed to the oral cavity. Furthermore, in the control group, the graft was not protected and remained exposed to the oral cavity which may cause physical loss of the grafted material or infection, contributing to the difference between the test and the control group; however, we aimed to examine if protecting the grafted material is necessary in these cases. Further studies may include another control group protecting the graft with primary closure. Lastly, performing CBCT right after the grafting and comparing it to the 12-month follow-up image may provide more accurate data. Nevertheless, the study employed one blinded examiner to minimize bias.

The innovative method employed to compare initial and final socket volumes holds promise for broader applications and warrants further exploration. By providing a precise three-dimensional evaluation of ridge volumetric changes, this technique allows for more accurate comparisons of contraction or expansion across different methods and biomaterials. Its objective nature eliminates the reliance on operator-dependent visual assessments and measurements, enabling a more comprehensive and unbiased analysis. Although the benefits of non-resorbable membranes in guided bone regeneration are well documented, their use in scenarios involving intentional exposure remains underexplored. This study addresses this gap by assessing volumetric changes in the alveolar ridge when d-PTFE membranes are combined with immediate implant placement and xenogenous bone grafting. Through tomographic evaluation, the study offers a detailed and objective understanding of the clinical implications of this approach.

Beyond advancing clinical knowledge, the findings of this study have significant practical value. The method evaluated here presents a cost-effective and reliable solution for routine clinical practice, minimizing the need for additional surgical procedures while achieving favorable outcomes. By addressing a critical gap in the literature and providing robust evidence for a simplified and efficient technique, this research has the potential to shape future guidelines and best practices for alveolar ridge preservation and immediate implant placement.

## 5. Conclusions

Based on our results, the alveolar-preservation technique using d-PTFE membranes intentionally exposed to the oral environment reduced alveolar bone contraction compared to using a bone substitute alone in post-extraction sockets with immediate implant placement. This clinical feature is important to preserve the buccal–lingual dimensions and avoid esthetic complications for further implant-supported restoration.

## Figures and Tables

**Table 1:** Demographic data of the evaluated patients. Mann–Whitney test (*p* > 0.05) and chi-square test (*p* > 0.05).

	*Control*	*d-PTFE*
***Gender (m/f)***	21:30	18:33
***Age (years): mean ± SD (Min–Max)***	44 ± 11 (26−60)	45 ± 9.4 (28−59)
***Tooth position***		
Molar	16	12
Non-molar	35	39
***Cause of tooth extraction***		
Fractures	24	21
Extensive caries	12	14
Endodontic failures	10	11
Root resoprtion	5	5

**Table 2:** Tomographic variable measurements at baseline (T0) and 12 months after surgical procedure (T12) for d-PTFE and control, respectively. Mann–Whitney U test, *p* < 0.05.

	*d-PTFE*		*Control*		
	Mean + SD	Range (Min–Max)	Mean + SD	Range (Min–Max)	p-Value
***Vol T0 (mm^3^)***	1510	1201–1793	1548	1271–1799	0.232
***Vol T12 (mm^3^)***	1446	1135–1769	1306	1102–1534	0.001
***% Contraction***	−4.3	0.97–(−8)	−16.0	−9.4–(−21)	<0.0001

## Data Availability

The data that support the findings of this study are available from the corresponding author upon reasonable request.

## References

[1] Araújo M.G., Lindhe J. (2005). Dimensional Ridge Alterations Following Tooth Extraction. An Experimental Study in the Dog. J. Clin. Periodontol..

[2] Mayer Y., Zigdon-Giladi H., Machtei E.E. (2016). Ridge Preservation Using Composite Alloplastic Materials: A Randomized Control Clinical and Histological Study in Humans. Clin. Implant Dent. Relat. Res..

[3] Schropp L., Wenzel A., Kostopoulos L., Karring T. (2003). Bone Healing and Soft Tissue Contour Changes Following Single-Tooth Extraction: A Clinical and Radiographic 12-Month Prospective Study. Int. J. Periodontics Restor. Dent..

[4] Fotek P.D., Neiva R.F., Wang H.-L. (2009). Comparison of Dermal Matrix and Polytetrafluoroethylene Membrane for Socket Bone Augmentation: A Clinical and Histologic Study. J. Periodontol..

[5] Al-Hezaimi K., Rudek I., Al-Hamdan K.S., Javed F., Nooh N., Wang H.-L. (2013). Efficacy of Using a Dual Layer of Membrane (dPTFE Placed over Collagen) for Ridge Preservation in Fresh Extraction Sites: A Micro-Computed Tomographic Study in Dogs. Clin. Oral Implant. Res..

[6] Araújo M.G., Sukekava F., Wennström J.L., Lindhe J. (2005). Ridge Alterations Following Implant Placement in Fresh Extraction Sockets: An Experimental Study in the Dog. J. Clin. Periodontol..

[7] Bartee B.K. (1998). Evaluation of a New Polytetrafluoroethylene Guided Tissue Regeneration Membrane in Healing Extraction Sites. Compend. Contin. Educ. Dent. Jamesburg NJ 1995.

[8] Cardaropoli G., Araújo M., Hayacibara R., Sukekava F., Lindhe J. (2005). Healing of Extraction Sockets and Surgically Produced—Augmented and Non-Augmented—Defects in the Alveolar Ridge. An Experimental Study in the Dog. J. Clin. Periodontol..

[9] Cardaropoli D., Tamagnone L., Roffredo A., Gaveglio L., Cardaropoli G. (2012). Socket Preservation Using Bovine Bone Mineral and Collagen Membrane: A Randomized Controlled Clinical Trial with Histologic Analysis. Int. J. Periodontics Restor. Dent..

[10] Paolantonio M., Dolci M., Scarano A., d’Archivio D., di Placido G., Tumini V., Piattelli A. (2001). Immediate Implantation in Fresh Extraction Sockets. A Controlled Clinical and Histological Study in Man. J. Periodontol..

[11] Botticelli D., Berglundh T., Lindhe J. (2004). Hard-Tissue Alterations Following Immediate Implant Placement in Extraction Sites. J. Clin. Periodontol..

[12] Francisco H., Marques D., Pinto C., Aiquel L., Caramês J. (2021). Is the Timing of Implant Placement and Loading Influencing Esthetic Outcomes in Single-Tooth Implants?-A Systematic Review. Clin. Oral Implant. Res..

[13] Araújo M.G., Wennström J.L., Lindhe J. (2006). Modeling of the Buccal and Lingual Bone Walls of Fresh Extraction Sites Following Implant Installation. Clin. Oral Implant. Res..

[14] De Rouck T., Collys K., Cosyn J. (2008). Immediate Single-Tooth Implants in the Anterior Maxilla: A 1-Year Case Cohort Study on Hard and Soft Tissue Response. J. Clin. Periodontol..

[15] Kan J.Y.K., Rungcharassaeng K., Morimoto T., Lozada J. (2009). Facial Gingival Tissue Stability after Connective Tissue Graft with Single Immediate Tooth Replacement in the Esthetic Zone: Consecutive Case Report. J. Oral Maxillofac. Surg. Off. J. Am. Assoc. Oral Maxillofac. Surg..

[16] Meijer H.J.A., Slagter K.W., Vissink A., Raghoebar G.M. (2019). Buccal Bone Thickness at Dental Implants in the Maxillary Anterior Region with Large Bony Defects at Time of Immediate Implant Placement: A 1-Year Cohort Study. Clin. Implant Dent. Relat. Res..

[17] Slagter K.W., Raghoebar G.M., Bakker N.A., Vissink A., Meijer H.J.A. (2017). Buccal Bone Thickness at Dental Implants in the Aesthetic Zone: A 1-Year Follow-up Cone Beam Computed Tomography Study. J. Cranio-Maxillofac. Surg. Off. Publ. Eur. Assoc. Cranio-Maxillofac. Surg..

[18] Araújo M.G., Lindhe J. (2009). Ridge Alterations Following Tooth Extraction with and without Flap Elevation: An Experimental Study in the Dog. Clin. Oral Implant. Res..

[19] Fickl S., Zuhr O., Wachtel H., Bolz W., Huerzeler M.B. (2008). Hard Tissue Alterations after Socket Preservation: An Experimental Study in the Beagle Dog. Clin. Oral Implant. Res..

[20] Novaes A.B., Suaid F., Queiroz A.C., Muglia V.A., Souza S.L.S., Palioto D.B., Taba M., Grisi M.F.M. (2012). Buccal Bone Plate Remodeling after Immediate Implant Placement with and without Synthetic Bone Grafting and Flapless Surgery: Radiographic Study in Dogs. J. Oral Implantol..

[21] de Carvalho Formiga M., Dayube U.R.C., Chiapetti C.K., de Rossi Figueiredo D., Shibli J.A. (2019). Socket Preservation Using a (Dense) PTFE Barrier with or without Xenograft Material: A Randomized Clinical Trial. Materials.

[22] Dahlin C., Linde A., Gottlow J., Nyman S. (1988). Healing of Bone Defects by Guided Tissue Regeneration. Plast. Reconstr. Surg..

[23] Barber H.D., Lignelli J., Smith B.M., Bartee B.K. (2007). Using a Dense PTFE Membrane without Primary Closure to Achieve Bone and Tissue Regeneration. J. Oral Maxillofac. Surg. Off. J. Am. Assoc. Oral Maxillofac. Surg..

[24] Hoffmann O., Bartee B.K., Beaumont C., Kasaj A., Deli G., Zafiropoulos G.-G. (2008). Alveolar Bone Preservation in Extraction Sockets Using Non-Resorbable dPTFE Membranes: A Retrospective Non-Randomized Study. J. Periodontol..

[25] Bartee B.K. (2001). Extraction Site Reconstruction for Alveolar Ridge Preservation. Part 2: Membrane-Assisted Surgical Technique. J. Oral Implantol..

[26] Barboza E.P., Stutz B., Mandarino D., Rodrigues D.M., Ferreira V.F. (2014). Evaluation of a Dense Polytetrafluoroethylene Membrane to Increase Keratinized Tissue: A Randomized Controlled Clinical Trial. Implant Dent..

[27] Bartee B.K. (1995). A Membrane and Graft Technique for Ridge Maintenance Using High-Density Polytetrafluoroethylene Membrane (n-PTFE) and Hydroxylapatite: Report of Four Cases. Tex. Dent. J..

[28] Dayube U.R.C., Furtado T.S.M., Formiga M.d.C., Shibli J.A. (2021). Intentionally Exposed D-PTFE Membrane and Guided Bone Regeneration after Malpositioned Dental Implant in Anterior Area: A Case Report with 1-Year Follow-Up. J. Int. Acad. Periodontol..

[29] Ronda M., Rebaudi A., Torelli L., Stacchi C. (2014). Expanded vs. Dense Polytetrafluoroethylene Membranes in Vertical Ridge Augmentation around Dental Implants: A Prospective Randomized Controlled Clinical Trial. Clin. Oral Implant. Res..

[30] Barboza E.P., Stutz B., Ferreira V.F., Carvalho W. (2010). Guided Bone Regeneration Using Nonexpanded Polytetrafluoroethylene Membranes in Preparation for Dental Implant Placements—A Report of 420 Cases. Implant Dent..

[31] Papi P., Di Murro B., Tromba M., Passarelli P.C., D’Addona A., Pompa G. (2020). The Use of a Non-Absorbable Membrane as an Occlusive Barrier for Alveolar Ridge Preservation: A One Year Follow-Up Prospective Cohort Study. Antibiotics.

[32] Zafiropoulos G.-G., Kačarević Z.P., Qasim S.S.B., Trajkovski B. (2020). Open-Healing Socket Preservation with a Novel Dense Polytetrafluoroethylene (dPTFE) Membrane: A Retrospective Clinical Study. Medicina.

[33] Rakhmatia Y.D., Ayukawa Y., Furuhashi A., Koyano K. (2013). Current Barrier Membranes: Titanium Mesh and Other Membranes for Guided Bone Regeneration in Dental Applications. J. Prosthodont. Res..

[34] Carbonell J.M., Martín I.S., Santos A., Pujol A., Sanz-Moliner J.D., Nart J. (2014). High-Density Polytetrafluoroethylene Membranes in Guided Bone and Tissue Regeneration Procedures: A Literature Review. Int. J. Oral Maxillofac. Surg..

[35] Sanz M., Lindhe J., Alcaraz J., Sanz-Sanchez I., Cecchinato D. (2017). The Effect of Placing a Bone Replacement Graft in the Gap at Immediately Placed Implants: A Randomized Clinical Trial. Clin. Oral Implant. Res..

[36] Chen S.T., Darby I.B., Reynolds E.C. (2007). A Prospective Clinical Study of Non-Submerged Immediate Implants: Clinical Outcomes and Esthetic Results. Clin. Oral Implant. Res..

[37] Assaf J.H., Zanatta F.B., de Brito R.B., França F.M.G. (2013). Computed Tomographic Evaluation of Alterations of the Buccolingual Width of the Alveolar Ridge after Immediate Implant Placement Associated with the Use of a Synthetic Bone Substitute. Int. J. Oral Maxillofac. Implant..

[38] MacBeth N., Mardas N., Davis G., Donos N. (2024). Healing Patterns of Alveolar Bone Following Ridge Preservation Procedures. Clin. Oral Implant. Res..

